# S100A6 and Its Brain Ligands in Neurodegenerative Disorders

**DOI:** 10.3390/ijms21113979

**Published:** 2020-06-01

**Authors:** Anna Filipek, Wiesława Leśniak

**Affiliations:** Nencki Institute of Experimental Biology, Polish Academy of Sciences, 3 Pasteur Street, 02-093 Warsaw, Poland; w.lesniak@nencki.edu.pl

**Keywords:** S100A6, CacyBP/SIP, Sgt1, neurodegeneration, β amyloid plaques, neurofibrillary tangles, Lewy bodies

## Abstract

The S100A6 protein is present in different mammalian cells and tissues including the brain. It binds Ca^2+^ and Zn^2+^ and interacts with many target proteins/ligands. The best characterized ligands of S100A6, expressed at high level in the brain, include CacyBP/SIP and Sgt1. Research concerning the functional role of S100A6 and these two ligands indicates that they are involved in various signaling pathways that regulate cell proliferation, differentiation, cytoskeletal organization, and others. In this review, we focused on the expression/localization of these proteins in the brain and on their possible role in neurodegenerative diseases. Published results demonstrate that S100A6, CacyBP/SIP, and Sgt1 are expressed in various brain structures and in the spinal cord and can be found in different cell types including neurons and astrocytes. When it comes to their possible involvement in nervous system pathology, it is evident that their expression/level and/or subcellular localization is changed when compared to normal conditions. Among diseases in which such changes have been observed are Alzheimer’s disease (AD), amyotrophic lateral sclerosis (ALS), epileptogenesis, Parkinson’s disease (PD), Huntington’s disease (HD), and others.

## 1. Introduction 

**Figure 1:**
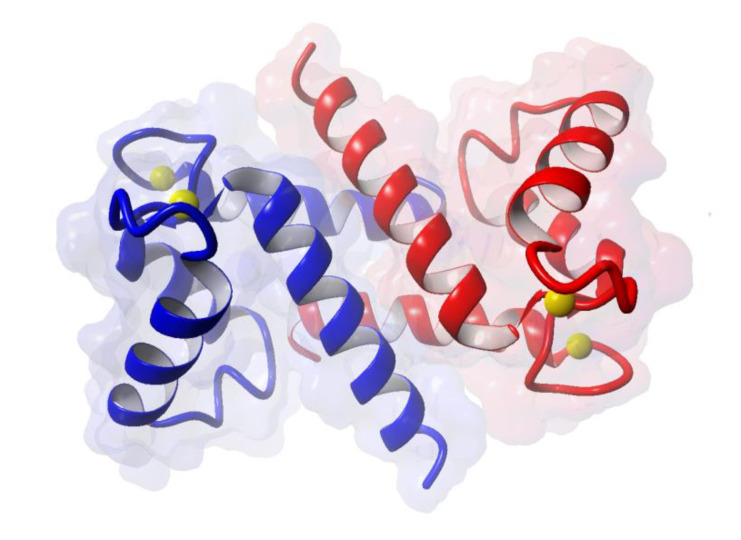
Structure of S100A6 dimer loaded with 4 Ca^2+^ (Protein Data Bank id:1K96) [3]. Each color, blue and red, represents one monomer; yellow balls represent Ca^2+^.

S100A6 is a low-molecular-weight Ca^2+^-binding protein of the S100 family [1,2]. It functions as a homodimer mainly formed by non-covalent interactions (Figure 1) and binds two Ca^2+^ per monomer, each through an EF-hand structure consisting of two α helices linked by a short loop region. 

Conformational change, evoked by Ca^2+^ binding, leads to exposure of hydrophobic surfaces involved in the interaction with ligands. Apart and independently of Ca^2+^, S100A6 can bind two Zn^2+^ per monomer with an affinity that enables it to act as a Zn^2+^ “chelator” both in the cytoplasm and outside the cell [4]. S100A6 is present in different mammalian cells and tissues, among them in the brain [5,6] where it is present in neurons and astrocytes [6,7]. Within the cell, S100A6 is localized mainly in the cytoplasm but, upon increase in intracellular Ca^2+^ concentration, may also associate with the nuclear envelope and plasma membrane [1]. Interestingly, S100A6 can be secreted and was found in extracellular matrix and various body fluids [8].

S100A6 interacts with numerous protein ligands both in the cell and extracellularly, which implicates its involvement in many signaling pathways. Interactions with almost all ligands identified so far depend on Ca^2+^ concentration, so the protein may confer Ca^2+^ sensitivity to other molecules and, in consequence, to different cellular processes. Extracellularly, S100A6 interacts with proteins such as lumican, PRELP (proline-arginine-rich end leucine-rich repeat protein), and IGFBP-1 (insulin-like growth factor-binding protein-1) [9] and with two membrane proteins, RAGE (receptor for advanced glycation end products) [10] and integrin β1 [11]. Through interaction with integrin β1, S100A6 activates intracellular signaling pathways involving integrin-linked kinase (ILK), focal adhesion kinase (FAK), and p21-activated kinase (PAK), and affects cell adhesion and proliferation [12]. 

Early identified S100A6 intracellular ligands include: GAPDH (glyceraldehyde-3-phosphate dehydrogenase), annexins (II, VI and XI), lysozyme, and cytoskeletal proteins such as tropomyosin, caldesmon, calponin, and tubulin. Quite recently, FOR20 (FOP-related protein of 20 kDa), a protein possibly involved in cilia formation, has been identified as a S100A6 ligand [13]. Binding to these ligands suggests that S100A6 plays a regulatory role in cytoskeleton organization and in membrane dynamics [1]. 

Another group of S100A6 ligands includes transcription factors of the p53 family, namely p53, p63, and p73, and a ubiquitin ligase, MDM2 (mouse double minute 2) [1]. This group of ligands implicates S100A6 in regulation of the stability/activity of p53 family transcription factors and, in consequence, in transcription. Of note, S100A6 binding to p53 affects p53 tetramerization and interferes with p53 binding to MDM2 ubiquitin ligase [14], and p300 acetyltransferase [15]. S100A6 also interacts with lamin A/C [16], a nuclear protein involved in chromatin organization, and with a nuclear transporter, importin α [17]. 

Research conducted by many laboratories concerning S100A6 function revealed a structurally similar but functionally diverse group of S100A6 ligands containing TPR and/or CS domains. This group includes melusin, kinesin light chain, Sgt1 (suppressor of G2 allele of Skp1), Hop (Hsp90/Hsp70-organizing protein), Tom70 (translocase of outer mitochondrial membrane 70), FKBP52 (peptidyl prolyl cis/trans isomerase FK506-binding protein 4), FKBP38 (peptidyl prolyl cis/trans isomerase FK506-binding protein 38), CyP40 (cyclophilin 40), CHIP (C-terminus of Hsc70-interacting protein), PP5 (protein phosphatase 5), and CacyBP/SIP (calcyclin binding protein/Siah-1 interacting protein) [1]. Interaction with these ligands may suggest involvement of S100A6 in cellular stress response, which seems to play a fundamental role in signaling pathways linked to various pathologies including neurodegeneration. 

Taking into account the presence of S100A6 and its two ligands, CacyBP/SIP and Sgt1, in the brain, in this review, we focus on the expression/localization of these proteins in various brain structures and on their possible involvement in neurodegenerative diseases. 

## 2. Expression of S100A6 in the Brain

Early immunohistochemical studies performed on rat brain slices showed that S100A6 is localized in neurons and astrocytes of various brain structures [6,7]. Particularly, the protein was found in pyramidal neurons of the hippocampus and cortex, granule cells in the cerebellum, neurons in the brain stem, in olfactory receptor cells, astrocytes in white matter, some ependymal cells (especially around the central canal), and in Schwann cells. Other studies performed on mouse brain showed high expression of S100A6 in astrocytes localized in the border zones of all brain ventricles, tanycytes of the hypothalamus, and neurons of the olfactory bulb, hippocampus, thalamus, cerebral cortex, brainstem, and cerebellum [18,19]. Interestingly, an increase in S100A6 reactivity in astrocytes of the mouse hippocampus was observed in aged animals [20]. As to the human brain, S100A6 immunoreactivity was detected in the entorhinal and occipital cortex, and in pyramidal neuron- and glial-like cells of the hippocampus; the hippocampal staining, however, became weaker with age [21].

Notably, S100A6 expression was observed in the two locations that harbor neural stem cells (NSCs) responsible for adult neurogenesis, that is, the subgranular zone (SGZ) of the dentate gyrus and the subventricular zone (SEZ) of the lateral ventricles. In the SGZ, high S100A6 expression was observed in quiescent NSCs and in lineage-restricted astrocyte precursors but only rarely in neural-lineage precursors [22]. The presence of S100A6 in the progenitor cells in SGZ was later confirmed by co-immunostaining with transcription factor, SOX2 [18]. In SEZ, S100A6 staining extended to the rostral migratory stream and was observed in NSCs but not in doublecortin-positive neuroblasts [23]. Expression of S100A6 in quiescent NSCs in SEZ was also detected by single-cell RNA sequencing [24].

## 3. Changes in S100A6 Expression in Neurodegenerative Diseases

### 3.1. S100A6 and Alzheimer‘s Disease (AD)

The most common neurodegenerative brain disorder is Alzheimer’s disease (AD). Symptoms of this disease are manifested by progressive memory deficits, cognitive impairment, and personality changes. The neuropathological hallmarks of AD include the presence of β amyloid plaques (also called senile plaques) in the extracellular space and of tau protein deposits (NFTs) inside neurons [25,26]. It is well known that the level of Ca^2+^ and activity of Ca^2+^-dependent signaling pathways are greatly implicated in AD [27,28]. Dysregulation of Ca^2+^ homeostasis and Ca^2+^-dependent signaling pathways leads to oxidative stress, mitochondrial disability, kinase and phosphatase dysfunction, cytoskeletal modifications, and finally, to synaptic loss, neuronal death, long-term depression, and memory loss. In addition, a proper homeostasis of other metal ions such as copper, zinc, iron, or manganese plays a crucial role in the pathogenesis of AD [29]. 

Published data reveal that extracellular β amyloid plaques present in the AD brain are surrounded by degenerative presynaptic endings, activated microglia, and reactive astrocytes [30,31,32]. Microglia express a range of different receptors, such as Toll-like receptors (TLRs), that can bind β amyloid plaques. Engagement of these receptors induces the release of tumor necrosis factor α (TNFα) and *interleukin 1β* (IL-1β), triggers neuroinflammation, and leads to neuronal damage. Reactive astrocytes surrounding β amyloid plaques were also found to be responsible for neuronal loss. Both activated microglia and reactive astrocytes, apart from neurotoxicity, were shown to be involved in the clearance of β amyloid plaques. 

Up to now, several reports regarding the possible involvement of S100A6 in AD have been published. S100A6 was found to be up-regulated in the brain of AD patients and of a mouse transgenic model of AD [33]. In particular, the S100A6 level was higher within the white matter (e.g., corpus callosum and internal capsule), in the hippocampal formation, and in the amygdaloid nucleus. Interestingly, almost all S100A6 immunoreactivity was found in reactive astrocytes surrounding β amyloid deposits. Some other studies have shown that chronic exposure of APP/PS1 (amyloid precursor protein/presenilin 1) double-transgenic mice (AD model) to Zn^2+^ increased Aβ deposition and S100A6 expression [34]. Both these phenomena could be reversed by applying a Zn^2+^ chelator, clioquinol. Exogenous S100A6 was found to reduce the level of protein deposits in APP/PS1 mouse brain sections and to protect cultured COS-7 cells against Zn^2+^ toxicity. Another work has shown that in the brain of APP23 mice (AD model), S100A6 was present both in the peripheral and central region of β amyloid plaques [19]. As Zn^2+^ co-localizes with senile plaques in AD patients and there is evidence that AD-related cognitive decline depends on the level of extracellular Zn^2+^ [35], the ability of S100A6 to bind Zn^2+^ may, similarly as in the case of S100B [36], prevent Zn^2+^-induced toxicity. All in all, presented data point out that in AD pathology, there is a correlation between S100A6 and amyloid plaques, reactive astrocytes, and Zn^2+^ level. Interestingly, a recent report revealed another aspect of S100A6 involvement in AD [37]. Namely, it was demonstrated that S100A6 enhanced the phosphatase activity of PPP5C toward phosphorylated tau protein. In agreement with studies described above are the results published by Wruck and coworkers [38] showing that in the AD gene signature, S100A6 is one of the most important proteins positively correlated with the AD phenotype. 

### 3.2. S100A6 and Other Neurodegenerative Diseases

Interestingly, differences in the expression pattern of S100A6 were observed in some other neurodegenerative diseases. For instance, in amyotrophic lateral sclerosis (ALS), S100A6 was found to be overexpressed within astrocytes surrounding the neurodegenerative lesions [39,40]. This up-regulation of S100A6 in ALS has been recently confirmed using LC-MS/MS and Western blot analysis [41]. ALS is a disease characterized by selective degeneration of motoneurons in the brainstem, spinal cord, and cortex [42]. In addition, atrophy of ventral roots, degeneration of the corticospinal tract, inclusions of aberrant neurofilament proteins in soma and axons, and astrogliosis can be observed [39]. As to astrogliosis, in patients suffering from ALS, it is mainly detected in the cerebral cortex and at the cervical and lumbar level of the spinal cord [43]. Of familial ALS cases, 20% are linked to mutations in superoxide dismutase (SOD1), which is activated by metal ions such as Zn^2+^. Thus, up-regulation of the Ca^2+^/Zn^2+^-binding protein, S100A6, within reactive astrocytes surrounding the neurodegenerative lesions in ALS may be linked to impaired Zn^2+^ homeostasis [40]. Interestingly, independently of Zn^2+^ binding, an in vitro assay has demonstrated that S100A6 can enhance SOD1 aggregation that contributes to ALS pathology [44].

An increase in S100A6 mRNA level was found in sclerotic hippocampi derived from epileptic patients [45]. In addition, S100A6 mRNA up-regulation has been observed in cortical regions of mouse brain in which epileptiform activity was induced by blood–brain barrier breakdown, albumin or transforming growth factor β1 (TGF-β1) [46], and following epilepsy-inducing traumatic brain injury [47]. Higher S100A6 expression has been later found in rat brain in a model of epilepsy induced by status epilepticus evoked by amygdala stimulation [48]. In particular, an increased S100A6 level was detected in the cortex and in the CA1 area of the hippocampus. Additionally, imaging analysis indicated that S100A6 expression was particularly high in reactive astrocytes. Moreover, the increase in S100A6 expression following epilepsy was shown to be widespread and long lasting. Increased S100A6 level has also been observed in the rat brain following kainic acid-induced epilepsy [20]. In particular, increased S100A6 immunoreactivity was found in astrocytes of the CA3 area of the hippocampus. Of note, in most cases, the up-regulation of S100A6 mRNA and/or protein level following epileptogenic stimuli coincided with marked neurodegeneration [20,46,47]. 

Degeneration of neurons is a major hallmark of traumatic brain injury (TBI). TBI is associated with very high disability and death rates. Many studies on the malfunction and neuron degeneration following TBI have revealed that Ca^2+^-overload, caused by mitochondrial dysfunction, is a common and an ultimate path to neuronal damage [49]. Using a rat model of TBI and different biochemical approaches, it has been shown that expression of S100A6 in the hippocampus was significantly diminished at 1–6 h post-injury, and then gradually returned to baseline after 14 days [50]. Changes in both protein and mRNA level were accompanied by animal cognitive deficits. This suggests that down-regulation of S100A6 is involved in early posttraumatic events that lead to secondary cognitive disorders while the elevation of S100A6 level in time is implicated in neuronal regeneration and repair. This possibility is supported by proteomic studies that showed increased S100A6 level in the spinal cord after injury [51]. Interestingly, down-regulation of S100A6 expression has been observed in the brainstem, hippocampus, and hypothalamus of mice subjected to the unpredictable stress paradigm [18]. This indicates that S100A6 might be involved in adaptive responses to stress probably via sensing the increased level of Ca^2+^ that results from the activity of stress-related brain structures.

In 2000, Hoyaux and coworkers suggested that S100A6 is involved in the inflammatory process, which could be triggered in corpora amylacea (C.A.) [52]. C.A. are cytoplasmic glycoproteinaceous inclusion bodies that accumulate in the human brain due to normal aging and, to a much larger extent, in AD and other neurodegenerative conditions. It has been found that the intensity of S100A6 immunostaining is noticeably high in C.A. This may be due to the accumulation of proteins associated with the cytoskeleton in C.A. [52]. Of note, as mentioned above, S100A6 interacts with cytoskeletal proteins such as caldesmon, calponin, tropomyosin, CacyBP/SIP, and tubulin [1].

## 4. Expression of CacyBP/SIP in Normal Brain and in Neurodegeneration 

One of the earliest targets of S100A6 identified and abundantly expressed in the brain is the CacyBP/SIP protein. This protein was originally discovered in Ehrlich ascites tumor cells and different mouse tissues [53,54] and later found in human cells [55]. In the brain, CacyBP/SIP is present in multiple structures, mainly in neurons and, to a lesser extent, in oligodendrocytes [56]. Recent results show that CacyBP/SIP is also present in neurons and oligodendrocytes of the spinal cord [57]. CacyBP/SIP interacts with many targets. Among them are cytoskeletal proteins such as tubulin, actin, and tropomyosin, but also tau and heat shock protein 90 (Hsp90) [58]. Of note, CacyBP/SIP possesses phosphatase activity [59] and also acts as a Hsp90 co-chaperone and exhibits chaperone activity itself [60]. All these interactions seem to have an impact on cell proliferation, differentiation, and cellular stress response, and in consequence, they may affect signaling pathways leading to brain development, aging, and/or neurodegeneration [58]. 

When it comes to aging and neurodegeneration, it was found that in neurons of 1) rat brain during physiological aging, 2) human AD brain, and 3) tauopathic mouse brain (a model of AD), CacyBP/SIP was present mainly in neuronal somata and co-localized with β-tubulin and tau protein [61,62]. Analysis of CacyBP/SIP localization in brain neurons during aging showed that in young animals, this protein was localized, similarly as tau, in both somata and neuronal processes. In aged rats, CacyBP/SIP and tau protein were found to be concentrated in cell bodies, and the tubulin staining pattern showed that the microtubule cytoskeleton was impaired. The change in CacyBP/SIP localization did not result from the loss of nerve fibers or from the loss of CacyBP/SIP [61]. These observations, together with data showing the up-regulation of CacyBP/SIP in neurons of patients suffering from another neurodegenerative disease, i.e., frontotemporal dementia (FTD) [41], point to an important role of this protein in AD-like pathology. This notion is corroborated by the observation that CacyBP/SIP can dephosphorylate tau protein [62]. 

Other neurodegenerative diseases, in which changes in CacyBP/SIP expression have been reported, include Parkinson’s disease (PD) and Huntington’s disease (HD). PD is a progressive neurodegenerative disorder, the second most common after AD. PD is characterized by both motor and non-motor symptoms. The appearance of motor symptoms is the consequence of gradual dopamine depletion followed by dysregulation of the dopaminergic pathway. In PD, dopaminergic dysregulation occurs due to selective loss of dopaminergic neurons (40%–60%) in the substantia nigra and accumulation of characteristic intracellular inclusions called Lewy bodies (LBs) [63]. LBs are found predominantly in the substantia nigra and serve as a major histopathological hallmark of PD; however, they are also found in other affected brain regions: Brainstem, basal ganglia, and cortex. Recent studies, with the use of mass spectrometry-based quantitative proteomics, have demonstrated decreased CacyBP/SIP expression in olfactory bulbs in PD patients [64]. 

HD is a hereditary neurodegenerative disease caused by expansion of a polyglutamine (polyQ) stretch in the huntingtin (HTT) protein. In the brain of HD patients, the mutant polyQ HTT is misfolded and forms toxic aggregates that are mainly present in neurons of the striatum. There is also a glial component of HD pathogenesis as activated microglia, reactive astrocytes, and oligodendroglia can be detected in many brain areas [65]. Clinical symptoms of HD include involuntary movements, impaired body balance, and a plethora of severe cognitive deficits. An approximately two-fold increase in CacyBP/SIP expression was observed in the striatum of a transgenic mouse model of HD [66]. 

Of note, in ALS, a neurodegenerative disease already described in connection with S100A6, the up-regulation of CacyBP/SIP could be detected by Western blot and LC-MS/MS analysis [41]. An increased CacyBP/SIP protein level was also found in selected brain structures such as the thalamus/hypothalamus, hippocampus, and brainstem of stressed mice [67]. The latter result suggests that CacyBP/SIP may lead to neurodegeneration through involvement in the cellular stress response. 

Another brain disorder in which the expression of CacyBP/SIP seems to be up-regulated is Down syndrome (DS) [68]. In particular, an increased level of this protein was detected in the brain of Ts1Cje mice (a DS model). In general, DS is characterized by mental and developmental retardation [69]. In most patients with DS, neuropathology such as reduced brain size and number of neurons, lower density of dendritic spines, impaired plasticity, and early onset of AD-like neurodegeneration is seen. Although it is known that this disease is caused by triplication of human chromosome 21, the proteins and molecular mechanism associated with DS symptoms remain unclear.

It is worth mentioning that global gene expression profiling using whole-genome microarrays has shown that CacyBP/SIP is up-regulated in another neurological disorder, that is, bipolar disorder (BD) [70]. BD is a severe neurological condition, the characteristic features of which include unusual shifts in: Mood, activity, concentration, and the ability to carry out day-to-day tasks [71]. The causes of BD are not fully understood and, thus, the possibilities of treatment of patients suffering from this disease are limited. 

## 5. Expression of Sgt1 in Normal Brain and in Neurodegeneration 

Among protein targets of S100A6 exhibiting high homology to CacyBP/SIP is a protein called Sgt1. Originally, Sgt1 was discovered in yeast cells (*S. cerevisiae)* as a protein that could be involved in activation of the CBF3 (centromere binding factor 3) kinetochore and the SCF (Skp, cullin, F-box) ubiquitin ligase complexes [72]. Later, it has been found that Sgt1 is a component of chaperone complexes as it binds Hsp90 and exhibits chaperone properties itself [73]. 

Studies regarding the expression of Sgt1 in mammalian tissues revealed its high level in the brain. In particular, the protein is present in Purkinje and glial cells of the white matter of the cerebellum and in neurons of the hippocampus and cortex [74]. Quantitative analysis of Sgt1-immunostained cells in the cortical regions of healthy aged versus AD brains showed a lower density of stained cells in the AD material. This suggests that Sgt1-immunopositive cells are selectively affected in certain cortical layers of the AD brain and that the protein might serve as a marker of neurons degenerating in AD. Recent work has shown that Sgt1 may be involved in PD and in dementia with Lewy bodies (DLB) [75]. The mRNA level of Sgt1 was found to be higher in the frontal and temporal cortex of PD and in substantia nigra of DLB brains. Although Sgt1 was not found in Lewy bodies, which are composed mainly of aggregated/phosphorylated α-synuclein, changes in the expression level of Sgt1 in PD and DLB suggest that this protein might be involved in the pathogenesis of these synucleinopathies. 

## 6. Summary and Conclusions

S100A6 is a Ca^2+^-binding protein belonging to the S100 family [1,2]. Members of this family are present in different cell and tissue types including the brain. The best known S100 protein present in the brain is S100B. In addition to being a Ca^2+^ sensor, S100B acts as a sensor and regulator of Zn^2+^ levels in the brain, and this metal-buffering activity is tied to its neuroprotective role and to inhibition of excitotoxicity [36].

The level of S100A6 in the brain is moderate in comparison to S100B, but it is expressed in various regions, which points to its role in brain functioning. S100A6 is present in neural stem cells and seems to be characteristic for the astrocytic lineage, but not exclusively, as many types of neurons are also S100A6-positive. The other two proteins, S100A6 ligands, are mainly neuronal. Multiple studies have documented that expression of all three proteins is altered in a diseased brain, especially in neurodegenerative disorders (Table 1), which suggests that they may be implicated in vital neural processes that become impaired in the diseased state. We are still far from being able to define the role of these proteins in the brain but, based on the accumulated data, it might be speculated that all three participate in cellular response to damage inflicted by metal ion dyshomeostasis, toxic protein aggregates, and other harmful factors/conditions associated with neurodegeneration.

The increase in the level of S100A6 in AD and its localization in astrocytes surrounding β amyloid plaques has been linked to its Zn^2+^-binding ability. Zn^2+^ is known to promote protein aggregation, and the presence of S100A6 may help to control local Zn^2+^ concentration or even to dissolve the toxic aggregates. Thus, data presented in this review open the way to the potential application of S100A6 as a marker of various neurodegenerative diseases and as a potential drug target. However, more research is required to further expand our knowledge regarding the role of S100A6 and its practical implications.

The S100A6 ligand, CacyBP/SIP, seems to be implicated in cytoskeletal reorganization that accompanies neurodegeneration or may affect protein phosphorylation, which has an impact on protein aggregation. Both CacyBP/SIP and Sgt1 may exert their chaperone/co-chaperone activities toward misfolded proteins that give rise to toxic oligomers and aggregates present in the brain of patients with different neurodegenerative diseases. To fully comprehend the importance of S100A6 and its two ligands, CacyBP/SIP and Sgt1, in brain functioning in norm and disease, more extensive studies are needed.

## Figures and Tables

**Table 1:** Changes in S100A6, CacyBP/SIP, and Sgt1 expression in neurodegenerative disorders.

Protein	Disease	Examined Region	Expression	Reference
S100A6	Alzheimer disease (AD)	- neocortex (white and gray matter) - prefrontal cortex - hippocampus	- immunoreactivity in astrocytes↑ - immunoreactivity in center and border zone of β amyloid plaques↑ - mRNA↑	[33,38]
	Amyotrophic Lateral Sclerosis (ALS)	- brainstem - spinal cord (dorsal root) - spinal cord (pyramidal tract)	- mRNA↑ - immunoreactivity in astrocytes↑ - protein ↑	[39,40,41]
	Epileptogenesis	- cortex	- mRNA ↑	[20,46,48]
		- hippocampus (CA1 and CA3)	- immunoreactivity in astrocytes↑	
	Traumatic brain injury (TBI)	- cerebral cortex	- mRNA↑	[47]
		- hippocampus	- mRNA↓	[50]
			- protein↓	
			- immunoreactivity	
			in pyramidal neurons↓	
	Stress	- cortex - brainstem - hippocampus - hypothalamus	- protein↓ - immunoreactivity in neurons and tanycytes↓	[18]
CacyBP/SIP	Alzheimer disease (AD)	- hippocampus	- protein – not changed	[62]
		- parieto-temporal cortex		
	Parkinson disease (PD)	- olfactory bulbs	- protein↓	[64]
	Huntington disease (HD)	- striatum	- mRNA↑	[66]
			- protein↑	
	Amyotrophic Lateral Sclerosis (ALS)	- non-motor cortex	- protein↑	[41]
		- spinal cord (pyramidal tract)	- protein↓	
	Stress	- thalamus/	- protein↑	[67]
		hypothalamus		
		- hippocampus		
		- brainstem		
	Down syndrome (DS)	- mouse embryo	- protein ↑	[68]
	Bipolar disorder (BD)	- lymphoblastoid cells	- mRNA ↑	[70]
Sgt1	Alzheimer disease (AD)	- cortex (temporal, angular, posterior cingulate)	- number of Sgt1 positive cells↓	[74]
	Parkinson disease (PD)	- temporal cortex	- mRNA↑	[75]
	Dementia with Lewy bodies (DLB)	- substantia nigra	- mRNA ↑	[75]

↑—increase; ↓—decrease.

## Abbreviations

AD	Alzheimer’s disease
ALS	Amyotrophic lateral sclerosis
C.A.	Suppressor of G2 corpora amylacea
CacyBP/SIP	Calcyclin (S100A6) binding protein/Siah-1 interacting protein
DS	Down syndrome
HD	Huntington’s disease
PD	Parkinson’s disease
Sgt1	Suppressor of G2 allele of Skp1
TBI	Traumatic brain injury.
